# “It is through body language and looks, but it is also a feeling” - a qualitative study on medical interns’ experience of empathy

**DOI:** 10.1186/s12909-019-1770-0

**Published:** 2019-09-04

**Authors:** Johanna von Knorring, Olof Semb, Martin Fahlström, Arja Lehti

**Affiliations:** Unit of Professional Development, Department of Clinical Sciences, Umeå University, SE-90187 Umeå, Sweden

**Keywords:** Empathy, Medical education, Professionalism

## Abstract

**Background:**

Empathy has long been recognized as a fundamental part of the professionalism of doctors and is considered to be both necessary and beneficial to doctor-patient relationships, although empathy is notoriously difficult to define and measure. Previous research on empathy has mostly consisted of quantitative studies measuring and evaluating empathy levels in students or medical residents. The aim of our qualitative study was to explore the lived experience of empathy among medical interns in Sweden.

**Method:**

We interviewed 16 medical interns, using semi-structured interviews. Content analysis was used to analyse the interviews.

**Results:**

The analysis led to the emergence of a main theme of empathy as being multifaceted and conflictual, consisting of descriptions (subthemes) of “being” and “doing”; of being uncontrollable and contextual; biased and situated and essential and conflictual. Since the components of empathy were also found to be interwoven, to provide a more holistic presentation of the results, we applied a socio-ecological model to the results inspired by Bronfenbrenner.

**Conclusions:**

We concluded that empathy is situated and contextual. By using the socioecological model empathy can be described as a systemic interaction between doctor and patient. Based on this we propose a more holistic approach to empathy in medical education to better prepare students for clinical practice.

## Background

Empathy has long been recognized as a fundamental part of professionalism for doctors [1], considered to be both necessary and beneficial to the doctor-patient relationship [2]. The benefits of empathy in medicine include better information transfer, enhanced compliance, faster recovery and increased patient participation in decision-making [3–7].

Previous research on empathy in the medical professional development has shown a decrease in medical students’ empathy as they proceeded through medical school [8–10] and Neuman et al. further conducted an extensive systematic review regarding the decline in empathy in the medical profession and its reasons. Despite varying results, an overall decline in empathy was reported [11]. Others have, on a similar vein, shown that empathy among medical residents is hindered by time restraints in the workplace [5]. A decline in empathy is not always reported, however. More recent studies have indicated a stability in empathy and even that it is improvable [4, 12, 13].

Despite being a much explored and evaluated phenomenon, there is still a lack of consensus on the definition of empathy [7, 14, 15]. In medical research, empathy is described in several ways. The neurobiological model of empathy includes activation of mirror neurons and complex neurobiological processes [16] In a more psychosocial model, empathy consists of three parts; (1) cognition – the ability to recognize and understand the patient’s emotion, (2) motivation – the motivation to communicate this understanding and (3) behaviour – the capacity to communicate understanding in an effective and helpful way [2, 9, 12, 17]. Other researchers state that empathy without an affective component is in fact not empathy [18, 19] but rather, detached concern [20]. Other models focus on for example the moral and relational aspects of empathy [21–23].

Hooker [24] argues that empathy as it has often been defined in medical research becomes too instrumental. In Hooker’s view, the key element of empathy is not simply that the doctors feel what the patient feels, but more importantly, that the doctors allow themselves to be affected by these feelings. Therefore, instead of a mere objective and/or instrumental understanding, the phenomenological perspective creates room for the existential and affective elements of the patient’s illness. This more authentic exchange of emotions will ultimately lead to trust and patient empowerment.

Having an empathetic approach in consultations is included in the curricula at almost all medical schools, and empathy is thereby seen as an accessible and teachable skill [25]. Although empathy has been found to be hard to define and difficult to measure, a large part of the earlier research on empathy have been quantitative studies trying to measure and evaluate levels of empathy in students or residents [9, 11]. However, there is a growing number of qualitative studies on empathy in the medical context [26]. In the light of Hookers formulation of empathy and a call for a broader understanding of empathy [24] it is important to study empathy in a frame of reference that allows greater complexity and to explore more aspects of empathy in the medical context. One way of exploring the complexity of empathy is to analyze the lived experience of empathy in a medical context.

In the search for a greater understanding of empathy, we aim to discuss empathy in the light of social constructivism and theoretical framework inspired by Bronfenbrenner, the renowned developmental psychologist, and his Human Ecology Theory [27]. The theory proposes that development unfolds in a set of systems involving cultural, social, economic, and political elements – not merely psychological (internal) ones. These systems and their interactions can facilitate or hinder development, central to the theory is the emphasis on person-context interrelatedness:

- The Microsystem: the individual most immediate surroundings – family, peers, school.
- The Mesosystem: interactions between the different microsystems.
- The Exosystem: links a social setting in which the individual does not have an active role and the individual’s immediate context e.g., local politics, neighbours. and
- The Macrosystem: cultural context e.g., socioeconomic status, and ethnicity.

## Method

The aim of this qualitative study was to explore the lived experience of empathy among medical interns in Sweden. The study is part of a larger project on empathy and doctors’ professionalism. The project was approved by the regional ethical review board in Umeå (Ref.No. 2016/50–31).

### Participants

Aiming towards variation in participants and breadth in the data, we invited medical interns, with no prior relation to any of the authors, from two university hospitals (one larger university hospital in a larger city and one smaller university in a smaller, provincial city) and one smaller hospital, assuming they have different experiences of working life. Differences in curricula can further be assumed between the university hospitals. Also, the burden of individual responsibilities for younger medical professionals can be assumed to vary between a smaller, provincial hospital and a larger university hospital (with more access to supervision for example).

All the medical interns were invited to participate via email and at a meeting, where a short presentation of the study was held. In total, 16 medical interns in Sweden agreed to participate and were interviewed about their experiences of empathy in working life.

Twelve of the 16 interviewees were working in a university hospital and four in a smaller hospital. Their ages ranged from 27 to 43. Seven of the interviewees were men and nine were women. Their experience of working as physicians after medical school before becoming medical interns ranged between three and 20 months. At the time of conducting the interviews, they had completed between 3 and 17 months of their internship, usually lasting for 18–21 months.

### Data collection

The 16 interviews were conducted between January 2014 and May 2015 by one of the authors (JVK). A semi-structured interview guide devised by three authors (JVK, AL, OS) was used to facilitate the participants’ discussion and thereby achieve richer content [28, 29] Example questions were: ‘What is empathy to you?’ or ‘Can you tell me about your experience of empathy in your daily work?’ When necessary, additional questions were asked to obtain more detailed responses. Each interview lasted about 40–80 min, in total 16 interviews. All medical interns participated voluntarily and received information about the study beforehand. Their informed consent was obtained and they were guaranteed full anonymity.

### Analysis

**Fig. 1:**
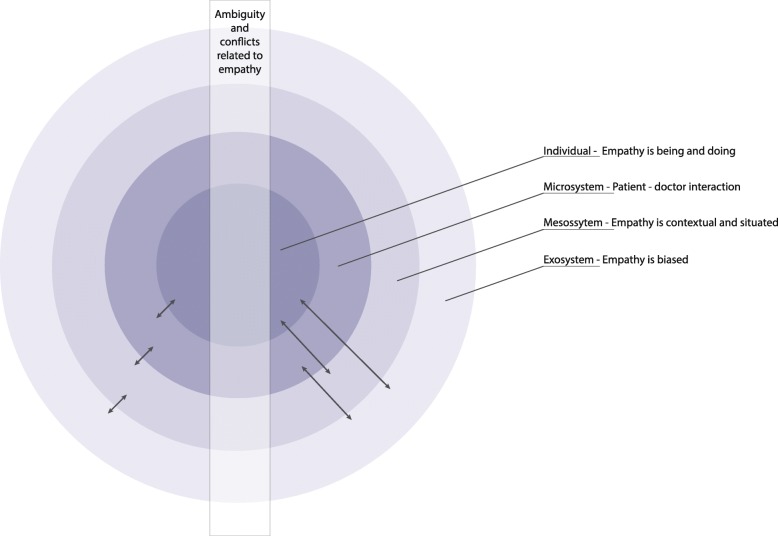
Model describing the different interacting components of empathy

Interviews were transcribed verbatim and then analysed with content analysis [30, 31]. With the starting point in qualitative content analysis (QCA) [30], the analysis started with reading, re-reading and the identifying of meaning units. The meaning units were then condensed and assigned codes close to the content. Codes were then organized into subcategories, which in turn were grouped into categories forming the manifest content. An example of the coding process is given in Table 1 below. These first steps were performed individually by two of the authors, JVK and AL. When there was consensus regarding the categories, together the three authors (JVK, AL and OS) re-read the transcripts as a whole in order to formulate an over-arching theme by interpreting the underlying meaning of the categories. This theme, together with the categories formed the results of this study. An overview of subcategories and categories is given in Fig. 1. At an early stage of the analysis, two interviewees were randomly selected and invited to share their opinions on the preliminary results. This was done to reduce the extent to which the prior assumptions and contextual pre-understanding of the authors would impact the analysis.

**Table 1:** Overview of subcategories and categories

Subcategories	Categories	Theme
Skill, personality trait, being empathetic, doing empathy, understanding, to feel with, automatic skill,	The quality of empathy	Empathy – a multifaceted and conflictual phenomenon
Time pressure, norms, values, communication, negative emotions, frustration, uncertainty, being powerless	Factors that affects empathy	
Level of engagement, lack of time, emergency situations	Conflicts in empathy

The first stage of data analysis was purely empirical, in accordance with QCA. The results from this analysis were diverse and multifaceted, prompting associations to Bronfenbrenner’s model [27]*.*

## Results

### Empathy: multifaceted and conflictual

Our analysis resulted in one overarching theme including three categories; conceptual ideas of empathy, factors that impact empathy, conflicts in empathy. The results show a broad and varied description of empathy, a salient feature in the daily work as a doctor. There are several factors described to impact empathy – it appears to be individual, situated as well as contextual which is further discussed in the light of Bronfenbrenner’s socioecological theory.

#### Empathy is being and doing

Empathy is described as a changeable *skill* susceptible to development and/or change – not simply a persistent *personality trait*. Empathy is assumed to be an important component of everyday work. It is also time-consuming and demands personal engagement from the doctors. To *understand* the patient’s context – cognitive – but also to *feel with* the patient – emotive – is described as essential for empathy.

*“A capacity to truly feel and understand another’s feelings and emotional state. Not simply to observe it but to really feel it.”*

*“To understand that a person is very stressed, for example; to then recognise this or draw attention to it, and then perhaps carry on more carefully, both when asking questions and attempting to calm the patient in the context of an examination.”*

Furthermore, the medical interns describe empathy as consisting of both an active *“doing empathy”* and a more passive *“being empathetic”* component. *Doing empathy* takes form via body language, communication and not avoiding difficult topics, for example drugs, abuse and loneliness. Doing empathy is also about establishing rapport and being able to convey one’s own empathy to the patient. *Being empathetic* is described as more intuitive, using the imagination to resonate with the feelings the patient displays, and to some extent “feel” the same thing as the patient. However, empathy is also described as occurring without any emotional engagement, instead it is a type of *automatic skill sets* in rendering an empathetic impression. This is sometimes considered to be necessary and situation-bound and occasionally more of a habit than a genuine response.

*“It is through body language and looks, but it is also a feeling; in some cases it feels more like learned behaviour than a genuine reaction. It becomes more a feeling, that the person does not feel entirely present. Yes, body language, looks, I suppose that's how it is”*

*“I may feel empathy for a person in a given situation, but it is not certain that the person understands this, as I have felt empathy but been unable to convey it, and I think this is quite important in your professional role – to be able to do this too – because otherwise it’s not worth quite as much.”*

Allowing one self to be guided by the emotions expressed by the patient, in order to empathize with the patient, is viewed as easy and natural, and, at the same time, as demanding and difficult. The difficulty is described as an *uncertainty* about how the emotions should be interpreted and that it is difficult to know how each individual would like to be treated. Additionally, the doctor’s’ own *negative emotions* such as irritation and frustration hinder empathy, in being able to feel empathetic as well as conveying the empathy felt for the patient. Angry, irritable and disrespectful patients as well as conflicts with the patient make it difficult to empathize. While, in contrast, it is easier to be empathic when meeting those in immediate crisis, sorrow, or social despair. Also described as challenging to the interns’ empathy were some patient groups in which the interns experienced lack of *communication* and trust – for example patients with psychiatric conditions, such as depression, dementia and addictions to alcohol or drugs. A lack of common ground in communication was described as creating a sense of powerlessness and frustration in the interns, hindering them from completely empathizing with the patient.

*“If the patient is disrespectful, grumpy or unpleasant to me, perhaps for some unknown reason, it can be difficult to have empathy and to engage with that person, even if you are still trying your best to be pleasant and be on your best behaviour.”*

*“Yes but if I think about how I myself mostly react in situations in close contact with the patient, normally if it is a person who is clearly unwell for some reason or is grieving or has had some kind of a crisis reaction, I think that I probably automatically feel empathy for that person, or that it is in some way easier to get close to a patient who is unwell.”*

*“You can still feel a bit powerless, if you can’t have a discussion, like you are not making any headway; you can suspect that a person is unwell but that they don’t want to say anything.”*

#### Empathy is uncontrollable and contextual

The medical interns describe a variety of factors that influence their empathy. A recurrent obstacle to empathy stated in the material is *lack of time*. The interns describe a conflict regarding the experience of not being able to be empathetic in their daily work with patients, even though they consider empathy to take only a small amount of time. They also describe *emergency situations* where they focus on parameters and medical measures, with empathy coming second. In times of stress and time pressure, the doctors’ agenda takes precedence and impairs the possibility to be present and guided by the feelings of the patient, thus complicating *communication*.

*“I think that factors such as a stressful work situation, brief contact with the patient, few beds and understaffing in all professional categories contribute to reduced empathy”*

*“you know that this is something we need to know, this is important for registering the patient, this is important for the medication; you’re also stressed and pressed for time – it's just chop, chop! – and you can end up sounding a bit like a robot. And the patient may want to get something off their chest, and so you feel that it’s probably something that’s very important for this person, but then we’ll need another two hours and we don’t have that time right now.”*

*“Sometimes you have patients in more of an emergency situation; then it’s more about keeping calm and getting the facts. There can also be a stressed friend or relative in the room, so you need to in some way show that you are both a kind-hearted person and competent.”*

#### Empathy is biased and situated

*Norms and values* are described as altering empathy. The attitudes of teachers and mentors during the educational years and in working life affect the intern’s’ self-perceived empathy, as do their own internal values and attitudes. Older colleagues act as role models and their manners and ability to be empathetic affect the interns’ engagement with the patients. It is easier to have empathy with those that one can relate to, for example based on age, gender, ethnicity, background and life situation.

“But I suppose that if the patient says something or there is something that strikes a chord with something you recognise, or for example, the person may have an appearance – that’s something I’ve experienced before – that reminds you of yourself or someone close. In those situations I’ve been able to feel – there’s just suddenly a lot more empathy.”

“If you hear from colleagues that yeah, that is so-and-so, they've been here a thousand times before and it’s just, yeah, it’s same old, same old. You then need to disregard that, start fresh and show that you are empathic and nice.”

#### Empathy is essential and conflictual

The factors that impact empathy generate conflicts in the daily work as a doctor. One of these conflicts is trying to find the proper *level of engagement*. There is an acceptance and appreciation of being affected and moved by the patient, but also a fear of becoming too involved and thereby being unable to provide good care. Another kind of conflict occurs when the doctor’s and the patient’s agenda differ. Sometimes, providing good care whilst simultaneously satisfying the patient is difficult, but on the other hand satisfying the patient is not always empathetic.

*“I think that if you make the wrong choices, you can really end up paying for it. It’s not especially nice to say so; it’s difficult to say it in a way that is perceived as empathic by the patient. I think that you can seem incredibly rude whatever you do. It perhaps doesn’t prevent me for feeling for that person, but it is difficult to show it. I probably become angry and upset in such situations.”*

## Discussion

The main finding of the study is that empathy, a multi-faceted concept of central importance in the patient-doctor relationship, is influenced by external as well as internal factors.

Most importantly, yet perhaps unsurprisingly, empathy varies within individuals as well as over different situations. The findings in our study are discussed in a model inspired by Bronfenbrenner’s socioecological model [27] where different components interact (Fig.1). This allows us to describe empathy as changeable, contextual and situated. Our model centers upon the individual’s lived experience, while simultaneously describing the interaction with the context. The context is described as different systems, or layers, between which interaction takes place. Further, the different systems also are interwoven.

Starting with the individual, the first layer focuses on the experienced features of empathy. The interviewees described empathy as simultaneously a personality trait and a skill. On the one hand, being empathetic is described as similar to being intuitive, to be able to resonate with, and to some extent, feel the same as the patient. On the other hand, empathy is also described as more of a conscious act, referred to as “doing empathy”, via body language, communication, and actively addressing difficult topics such as drug abuse. Some of the interviewees refer to empathy without emotional engagement as an automatic skill that renders an empathetic impression. This is sometimes considered necessary and situation-dependent; sometimes more of a habit. The many ways of describing empathy and its various components in our study is coherent with established concepts of empathy [32]. In accordance with earlier research we also notice that the experience of empathy seems to be broad and vague [11, 33]. Further, we find empathy to be a topic seldom reflected upon and the interview itself becomes an occasion for reflection and an opportunity for clarification of what empathy actually means to the interviewee.

The second layer shifts focus from the individual, towards how the doctors are influenced by, and interact with, the emotions of the patient. The interviewees saw their own negative emotions and conflicts with the patient as an obstacle to empathy. Allowing oneself to feel irritable or angry towards the patient is difficult, as it is in direct conflict with their ideal professional behavior. Halpern believes that this is common but the risk is that in the attempt to suppress non-accepted emotions, the doctor may overlook the patient’s perspective [21]. Negative emotions originating with the patient sometimes lead to a deliberate response, where the doctors, as a sort of defense on an intellectual plane create an understanding of the patient. Alternately negative emotions lead to more unconscious reactions, manifested as powerlessness and frustration. This is also described by Söndergaard as the transference of the patient’s emotions which, if not recognized and processed, can lead to that the doctor’s negative emotions, thoughts, and beliefs about the patient unconsciously being transferred on to the patient in the form of countertransference [34]. These reactions are liable to hinder good and efficient care for example by avoiding certain questions/topics, making unnecessary tests or doctors failing to focus on whatever is essential in meeting a particular patient [35].

In the third layer, empathy is described as contextual and influenced by immediate external factors surrounding the doctor as well as the patient. Our study confirms previous findings that lack of time, stressful work environments and lack of opportunity for face-to-face interaction are barriers to empathy [36]. We also notice that communication is of great importance both for being empathic, and in conveying that empathy to the patient.

The outer, fourth layer of our model describes the doctor and patient in a more complex interaction with the norms in healthcare and society in general. We find norms to be a powerful factor affecting empathy. This applies both to the doctor’s own values and the values outlined by the healthcare system and colleagues. For example, there is a conflict between the ideals of the young doctors and the behaviors and attitudes of their role models, or the general jargon in the clinic. This phenomenon is similar to what is often referred in education, such as the hidden curricula that describes informal norms and socialization processes in medicine [1, 37]. In our view, the discrepancy between official and hidden agenda may confuse and belittle the importance of empathy and professionalism. Empathy can therefore be experienced as optional instead of an integrated part of medicine. If that attitude can persist over time, it could lead to an unreflective and unprofessional attitude. We also find that norms affect the ability to relate to others, as it is easier to establish rapport with patients sharing the same socioeconomic status and educational background of the doctor. Our findings correspond with previous research [38] and highlight the risk for inequity in healthcare.

Interwoven in the model is the ambiguity and conflicts related to empathy. The interviewees experience empathy as something that require effort, combined with a fear of compassion fatigue. They describe a cumbersome process of finding a proper/sustainable level of involvement; not too tiresome yet with enough commitment to understand the patient and provide good patient-centered care.

What emerged in this study may be tainted by the local context; all participants conducted their university studies in Sweden. However, the participants represent a variation in hospital size and location, as well as educational institutions. Furthermore, the participants represent a variation in age and experience, whereas they are, at the same time, equally distributed by gender. As empathy is a complex phenomenon, we consider a qualitative method to be preferable. The qualitative content analysis renders a description close to the participants’ own words yet diverse and rich [30].

All of the authors have different backgrounds (psychology, family medicine, rehabilitation medicine and a junior doctor) which supposedly increases the reliability [39]. The fact that only one author did the interviews can be both an advantage and a disadvantage, it can provide a deeper understanding for the process and material in an early stage which can generate a richer material, but at the same time it might reproduce questionnaire-biases. Three of the authors have, to various extents, experiences of working as doctors. This made it easier to understand the context and situations, however the existing knowledge increases the risk of overlooking important findings that could benefit from further description.

A limitation of the study is that we did not include the macro perspective of Bronfenbrenner’s theory [27] because the data does not allow for this level of analysis. Future studies should also include questions that render data on the macro perspective to fully discuss the entire socioecological model.

## Conclusion

This paper adds to earlier research that empathy is a multifaceted and complex phenomenon, where the descriptions of empathy vary; yet at the core there is a will to understand another person as a whole, to convey that understanding with respect to interpersonal boundaries and differences. We emphasize that empathy is situated and contextual, influenced by time restraints, language and communication barriers as well as norms and attitudes. This contextual complexity can, in our view, explain the varied results of earlier research aimed at measuring changes in empathy. By using the socioecological model, a more systemic understanding of the doctor-patient relationship becomes salient. Doctors encounters not only patients, but also presumably the systems of the patients. The doctor-patients systems interact, but the doctors and patients also interact with the different levels of their own systems. Based on this we propose a more holistic approach in teaching empathy and professionalism in medical education by adding systemic knowledge to better prepare students for clinical practice.

## Data Availability

Supporting data can be obtained through contact with the corresponding author.
